# Strength of Association Between Smartphone and Social Media Screen Time with Dietary Behaviour and Physical Activity in United Arab Emirates Adults: A Cross-Sectional Study

**DOI:** 10.3390/nu18010067

**Published:** 2025-12-25

**Authors:** Mo’ath F. Bataineh, Sameera Koodakkadavath, Aleena Hassan, Hassan M. Al Marzooqi, Hanan S. Afifi, Mohamed G. Shehata, Habiba I. Ali

**Affiliations:** 1 Department of Nutrition and Health, College of Medicine and Health Sciences, United Arab Emirates University, Al Ain, United Arab Emirates; m.bataineh@uaeu.ac.ae (M.F.B.);; 2 Food Research Section, Applied Research and Capacity Building Division, Abu Dhabi Agriculture and Food Safety Authority (ADAFSA), Abu Dhabi, United Arab Emirates

**Keywords:** smartphone use, social media use, screen time, dietary habits, physical activity, obesity, UAE

## Abstract

Background: Smartphones and social media (SPSM) use has become an integral part of life around the globe, including the United Arab Emirates (UAE). This study investigated the association and strength of association between SPSM screen time and dietary and physical activity behaviours among adults in the UAE. Methods: A cross-sectional household-based study was conducted in the Emirate of Abu Dhabi, UAE, between June 2024 and March 2025. A total of 5152 adults aged 18 years and above were selected through a multistage stratified random sampling method. Data were obtained from structured interviews that collected information about demographics, SPSM use time, dietary habits and physical activity levels. The statistical analyses conducted using SPSS software. Results: The mean SPSM usage time was 277.6 ± 165.6 and 234.6 ± 170.8 min per day for smartphone and social media, respectively. Social networking was the main purpose for smartphone (71.7%) and social media (74.8%) usage. Higher SPSM use was significantly associated with more frequent consumption of unhealthy foods, including sugar-sweetened beverages, fast food, and energy drinks (*p* < 0.001). The associations between SPSM and dietary behaviour and physical activity were generally weak. Only social media usage time was significantly associated with physical activity (*p* = 0.012). Conclusion: SPSM use time is associated with dietary patterns and physical activity among adults in the UAE. Higher smartphone and social media screen time was associated with increased consumption of unhealthy foods and lower physical activity; however, the relationships were weak to moderate.

## 1. Introduction

Digital technology has changed our lives significantly in the last two decades, with smartphones becoming one of the most powerful technologies that have transformed the way people live today. Global Statistics show that by 2025, approximately 7.21 billion people, which accounts for 90% of the world population, will be smartphone users [1]. Moreover, around 6.04 billion (73.2%) are expected to have access to the internet, while 5.66 billion people (68.7%) will be active on social media platforms [2]. In line with this trend, the UAE is highly digitally connected, with 11.3 million internet users (99% penetration), 12.5 million social media identities (110% of the population), and 23 million active mobile connections (202% of the population) by the end of 2025 [3]. Smartphones have numerous benefits, including socialisation, information retrieval, entertainment, and time management [4,5]. In recent years, smartphones have been widely used to access multiple social media platforms. Social media are applications and websites that provide a digital environment for smartphone users to send or receive digital content [6]. They include social networking services such as Facebook, Instagram, TikTok, Twitter, and YouTube; messaging apps such as WhatsApp and Snapchat; online shopping apps; and educational or news websites.

The time spent on the internet and social media apps has increased significantly in recent years, with an average of 2 h and 27 min per day globally [7]. Thus, SPSM have started to change the users’ needs, values and interests and raised worries about overuse and addiction [8]. This change has become so profound that the idea of a “digital food environment” has been established [9]. The World Health Organization (WHO) defines digital food environment as the “online settings through which flows of services and information that influence people’s food and nutrition choices and behaviour are directed” [10]. However, the digital food environment can shape the individual’s dietary patterns in both positive and negative ways.

SPSM helps the food industry communicate with consumers about food availability through advertising, hashtags, and digital marketing analytics [11]. Studies show that digital food marketing exposes people to nutritional content on social media that further shapes dietary habits, often leading people to favour high-energy foods with low nutritional value, a trend further reinforced by social comparison and the normalisation of unhealthy eating behaviours [11,12]. Social media influencers have become an essential part of the food marketing industry, due to their accessibility and credibility [13]. They influence dietary habits by promoting food trends, normalising unhealthy eating habits, particularly among children, and using persuasive, engaging content to shape users’ food choices and purchasing behaviours [14,15]. Another application of SPSM is the widespread use of food delivery applications that promote convenient but unhealthy food options that have been reported to cause weight gain and health problems [16].

Due to the growing popularity and usage rates, numerous studies carried out on SPSM users showed a significant relationship between internet addiction, behavioural problems, restlessness, anger and disruption of duties [17,18,19]. This has been shown to affect the quality of life, affecting nutrition and physical activity [20,21]. Nutritional content on social media can further shape dietary habits, often leading people to favour high-energy foods with low nutritional value, a trend further reinforced by social comparison and the normalisation of unhealthy eating behaviours. Social media pressurises people to compare themselves to others, which makes them feel less confident and alone [22]. Recent reports also highlight the growing issue of social media fatigue among young adults in the UAE, a phenomenon where users feel mentally exhausted due to the continuous psychological and technological pressure associated with internet use [23].

Additionally, the UAE have one of the highest social media usage rates in the Middle East and North Africa (MENA) region, with a daily usage typically exceeding 3–5 h, mainly using social media apps [24]. At the same time, obesity rates in the UAE have turned into an alarming issue, with rates significantly higher than the global average [25]. This increase in obesity rates cannot be explained only by genetics [26], but seems to have a close association with other behavioural and environmental factors, such as too much screen time, too much ultra-processed food consumption and reduced physical activity [27,28]. Studies show that skipping breakfast has also been linked to increased time spent on SPSM [20]. Excess use of SPSM can also lead to increased sedentary life and thus reduced physical activity, which is further related to hormonal disproportion that encourages higher appetite and insulin insensitivity, which intensifies the danger of obesity [29,30].

Although associations between SPSM’s screen time and dietary habits have been reported in the UAE [31], this study collected data close to the COVID-19 pandemic, did not employ a structured sampling technique to obtain a representative sample, and did not assess the strength of the association. Therefore, there is a scarcity in research that has explored this interaction at a large-scale and household-based study, to ensure representativeness across resident adults. Our study also aimed to examine whether SPSM use may contribute to low levels of physical activity, which have been estimated to be below expected in previous studies among adults in the UAE [32]. Moreover, most studies in the literature have focused on these interactions among adolescents; consequently, there is a research gap for other age groups. Given the high levels of digital access and urbanisation, it is essential to examine how these conditions interact in the UAE. Moreover, none of the previous studies have investigated the strength of the relationship between SPSM and dietary behaviour and physical activity. Hence, the objective of this study was to examine the association and strength of association between screen time spent using SPSM, dietary behaviours, and physical activity levels among adults in the UAE. Based on the research questions, we hypothesised that higher SPSM screen time is associated with less healthy dietary behaviour and lower physical activity among adults in the UAE, and that these associations would be statistically significant.

## 2. Materials and Methods

This study used a cross-sectional design, and the study protocol was approved by the United Arab Emirates University Social Sciences Ethics Committee—Research (ERSC_2024_4427) and the United Arab Emirates University Human Research Ethics Committee (ERH_2023_2669_05). Participants received detailed information about the project and the purpose of the study, and each participant provided written informed consent to participate before data collection began. The study adhered to the STROBE (Strengthening the Reporting of Observational Studies in Epidemiology) reporting guidelines for cross-sectional research.

### 2.1. Study Population

The Abu Dhabi Agriculture and Food Safety Authority (ADAFSA), in collaboration with the College of Medicine and Health Sciences at the United Arab Emirates University (UAEU) and the Statistics Centre-Abu Dhabi (SCAD), has launched a comprehensive field survey aimed at determining food consumption rates and trends across Abu Dhabi, UAE. This survey is designed to gather current and accurate data on the individual food consumption rates within the various regions of the Emirate, including Abu Dhabi City, Al Ain, and the Al Dhafra region. It measured the types and quantities of foods consumed, analysed consumption patterns, and identified the cognitive, social, demographic, and environmental factors influencing these patterns. The participants in this study were adults randomly sampled for the Food Consumption Survey in the Emirate of Abu Dhabi.

This cross-sectional study included 5152 adults aged 18 years or older who were recruited from 3920 households randomly selected to form a representative sample of people residing in the Emirate of Abu Dhabi. The inclusion criteria of the study were the following: (1) Emirati and non-Emirati nationals who are permanent residents of the Abu Dhabi Emirates; (2) above 18 years of age on the day of the interview. Emirati and non-Emirati nationals not residing in the Abu Dhabi Emirate and/or children and adolescents were excluded from the analysis of this study.

### 2.2. Sample Size

A sample size calculation, based on a 99% confidence level, a 2.0% confidence interval, and an assumed population proportion of 50%, indicated the need to recruit a minimum of 3500 adult participants to obtain a representative sample from both UAE citizens and non-citizens residing in the Emirates of Abu Dhabi. To meet this requirement and allow for potential attrition rates or lack of response estimated as 30%, the recruitment target was set at approximately 5000 participants, with 1–2 individuals recruited per selected household. According to data provided by the Statistics Centre—Abu Dhabi (SCAD), the targeted household distribution across the three main regions was as follows: Abu Dhabi city—1750 households, Al Ain—1470 households, and Al Dhafra—700 households.

### 2.3. Sampling Protocol

Data about residents of selected neighbourhoods were obtained from SCAD using a proportionate multistage stratified random cluster sampling protocol. The Emirate of Abu Dhabi is divided into Al Ain, Abu Dhabi and the Al Dhafra region. Within each region, districts were randomly selected, followed by the random selection of areas. Within each selected area, a neighbourhood was randomly selected. And within each selected neighbourhood, households were randomly selected. Finally, participants were randomly chosen from each selected household.

### 2.4. Data Collection

Data collection was carried out by locally based field teams composed of 2–3 qualified and trained research team members, including recruiters and interviewers who had received training on how to conduct and collect information during household visits. During the field visits, the participants received detailed information about the project and the purpose of the study, and each participant gave their informed consent for participation prior to data collection. The obtained information included socio-demographic factors, physical measurements of weight and height and body composition, dietary patterns and social media and smartphone use.

### 2.5. Questionnaire

Socio-demographic data were collected through structured home visits conducted by trained interviewers adhering to a standardised protocol developed specifically for this study. A comprehensive, multi-section questionnaire was employed to obtain detailed information on socio-demographic variables and physical activity behaviours. Physical activity levels were assessed using the International Physical Activity Questionnaire–Short Form (IPAQ-SF) [33]. The validated Arabic version of the IPAQ-SF was used in this study. Accordingly, participants were subsequently classified into low, moderate, or high physical activity based on their responses as previously described [33].

Participants’ dietary behaviour was assessed using a set of ten items related to eating behaviours. These items captured the frequency of food and beverage intake over the past week. The questions covered the five main food groups (grains, vegetables, fruits, dairy, and proteins), as well as breakfast consumption, energy drinks, sugar-sweetened beverages, snacks, and fast food. All food-related items were rated on a 5-point scale, ranging from 0 (never) to 4 (daily).

Smartphone and social media usage time was assessed using the following questions separately: “How many hours per day did you use your smartphone for personal reasons (not for work) in the past 30 days?”. The questions regarding smartphone and social media usage time were categorised into weekdays and weekends, and the average daily hours of use obtained from weekdays and weekends were later calculated and reported as hours of weekly use. The purpose of using smartphones and social media were assessed separately using the following question: “What was the single most frequently used smartphone content type during the past 30 days?” participants were to select only one option from a list of 7 choices including education/information search, e-mail, social networking service (SNS) (e.g., Instagram, X (Twitter), Facebook), game, movie/video/music, online shopping, and others [34]. These self-reported values were then included in the subsequent analyses.

### 2.6. Assessment of Body Weight and Height and Calculation of Body Mass Index

Anthropometric measurements, including weight, height, and body fat percentage, were recorded, and Body Mass Index (BMI) was subsequently calculated. Weight and body fat were assessed using the Tanita BC-545N segmental body composition monitor (Tanita Corporation, Tokyo, Japan), while height was measured with the InBody Push portable stadiometer (InBody Co., Ltd., Seoul, Republic of Korea). The InBody Push is an advanced stadiometer equipped with an ultrasound sensor designed for precise height measurement. Height was recorded to the nearest 0.1 cm and weight to the nearest 0.1 kg, adhering strictly to the manufacturer’s guidelines. According to the WHO 2007 guidelines, BMI categories are defined as underweight (<18.5 kg/m^2^), normal weight (18.5–24.9 kg/m^2^), overweight (25.0–29.9 kg/m^2^), and obese (≥30.0 kg/m^2^) [35].

### 2.7. Statistical Analysis

All analyses were performed using SPSS version 31.0 (IBM, Armonk, NY, USA). Continuous variables were presented as means and standard deviations (SD), while categorical variables were presented as frequencies and percentages. To analyse differences in smartphone and social media use across dietary intake groups, ANCOVA analysis was conducted with the use of age, gender, monthly income and BMI as covariates. The analysis used the food frequency as the dependent variable and the smartphone and social media use time as independent variables. Additionally, chi-square analysis was used to assess the associations between categorical variables. The strength of association (effect size) between categorical variables was assessed using Cramer’s V, with associations classified as weak (<0.20), moderate (0.20–0.40), or strong (>0.40). Moreover, the non-parametric Kendall’s tau-b correlation coefficient was assessed. The *p*-value was taken to be statistically significant at *p* < 0.05.

## 3. Results

### 3.1. Demographics and Dietary Patterns

The demographic characteristics of the participants are shown in Table 1. Among the 5152 participants, 62.8% were females, 58.8% were Emirati nationals, and 66.0% were married. The mean age was 36.4 ± 12.0 years, and the mean BMI was 27.2 ± 5.2 kg/m^2^. Additionally, 68.4% of the participants reported having good perceived health. The majority of the participants (52.5%) reported having a college/university degree. The majority of the participants (63.4%) had low physical activity. The mean smartphone and social media usage time was 277.6 ± 165.6, and 234.6 ± 170.8 min per day, respectively. A large proportion (39%) of the participants reported using a smartphone for 4 to less than 6 h per day. The majority (34.6%) of the participants used social media for 2 h to less than 4 h per day.

The weekly food intake frequencies are listed in Table 2. The majority of participants reported consuming fruits daily (44.4%), vegetables daily (63.2%), dairy group daily (33.3%), meat daily (79.2%), and grains daily (85.8%). Moreover, a majority of the participants reported consuming breakfast daily (64.6%). For the energy drinks, 78.5% of participants reported never using them. A large proportion of participants reported consuming sweetened beverages one to two times a week (33.1%), consuming fast food one to two times a week (50.3%), and consuming snacks on a daily basis (52.3%).

### 3.2. Associations of SPSM Screen Time with Dietary Patterns and Physical Activity

Table 3 shows the average time spent on smartphones and social media (minutes/day) reported by the weekly frequency of consumption from food items. The ANCOVA analysis, which was adjusted for the covariates age, BMI, sex, and monthly income, revealed significant differences in both smartphone and social media usage time across the categories of weekly consumption for most of the food items except for grains and breakfast with smartphone usage time and meat with social media usage time.

Table 4 presents the associations between dietary behaviour and categories of smartphone and social media usage time. All tested food items showed significant associations: as smartphone time use increased, weekly consumption of unhealthy foods also increased. The strongest connections were observed for sugar-sweetened beverages with χ^2^ (1,12) value of 461.929, *p*-value < 0.001 and Cramer’s V of 0.173, fast food with χ^2^ (1,12) value of 405.021, *p*-value < 0.001 and Cramer’s V of 0.162, and energy drinks with χ^2^ (1,12) value of 220.790, *p*-value < 0.001 and Cramer’s V of 0.120. Even healthy foods, such as fruits, vegetables, and grains, showed statistically significant but weak associations, indicating that the impact of smartphone usage time on healthy food consumption habits was weak. The smartphone and physical activity showed a χ^2^ (1,6) value of 8.640, a *p*-value = 0.195, and a Cramer’s V value of 0.029, indicating a non-significant weak association between smartphone and physical activity.

Additionally, all tested food items showed significant associations with social media usage time. As the duration of use increased, weekly consumption of unhealthy foods also increased. The strongest connections were observed for sugar-sweetened beverages with χ^2^ (1,12) value of 619.416, *p*-value < 0.001 and Cramer’s V of 0.200, fast food with χ^2^ (1,12) value of 401.854, *p*-value < 0.001 and Cramer’s V of 0.161, and energy drinks with χ^2^ (1,12) value of 309.643, *p*-value < 0.001 and Cramer’s V of 0.142. Even healthy foods, such as fruits, vegetables, and grains, showed statistically significant but weak associations, indicating that the impact of social media time on healthy food consumption habits was weak. The social media and physical activity showed a χ^2^ (1, 6) value of 16.370, a *p*-value = 0.012, and a Cramer’s V value of 0.040, indicating a significant, weak association between social media and physical activity.

### 3.3. Associations of SPSM Main Use Purpose with Gender

Table 5 shows the association between the primary purpose to use smartphone and/or social media and gender. In general, social networking was the primary reason to use smartphones (71.7%), followed by movies/video/music (10.2%) among all participants. Similarly, social networking was the primary reason to use social media (74.8%), followed by news (7.6%) among all participants. The chi-square test revealed a weak significant association between smartphone use purpose and gender (χ^2^(1, 6) = 97.853, *p* < 0.001; Cramer’s V = 0.138). In addition, the chi-square test revealed a weak significant association between social media use purpose and gender (χ^2^(1, 6) = 91.131, *p* < 0.001; Cramer’s V = 0.133). Male participants reported higher smartphone use for movie/video/music, games, and e-mail, whereas females reported greater use for socialising and educational content. Additionally, Male participants reported higher social media use for socialising, and news apps, whereas females reported greater use for shopping.

## 4. Discussion

This study reports the association and strength of association between SPSM and dietary behaviours and physical activity levels in a large household study of adults in the Abu Dhabi Emirate of the UAE. The results of our study show that the majority of the participants reported using SPSM for more than 2 h per day. This was slightly higher than the rate reported in a previous study in the UAE [31]. Indeed, current results show that the average smartphone use time was approximately 277.6 min per day and social media use was approximately 234.6 min per day, which also supports the fact that most of the smartphone users in the Middle East and Africa (MENA) region spend more than three hours per day on social media, which is above the global average [36]. This is of great concern because studies show that people who spend more than recommended hours of less than thirty minutes on SPSM are more prone to mental health issues [37,38]. This high level of social media use demonstrates how deeply integrated digital platforms have become into daily life and, by extension, the food environment.

Our study indicates that most participants primarily used SPSM for socialising, with fewer using it for study purposes or for health information, and the smallest proportion of participants used it for dietary advice. This trend aligns with global findings, in which social media and socialising account for most of the time spent on digital devices, which emphasises the likelihood of SPSM as sources of passive information consumption and behavioural reinforcement, rather than health promotion or education [39,40]. One reason might be that users find dietary advice and health information on social media less trustworthy or engaging than other content [41]. Furthermore, the marketing industry mainly concentrates on advertisements and entertainment, which capture most social media attention, making health- and nutrition-related information less visible [42]. The current study showed that males reported higher use of entertainment and news apps. In contrast, females showed slightly greater engagement with socialising and educational content, consistent with findings in the literature, which indicate that men use digital platforms primarily for information seeking and entertainment. In contrast, women use them for maintaining relationships and social interaction [43].

Impulsivity may underline this increased use of SPSM, as individuals tend to seek instant gratification, respond more quickly to rewarding stimuli, and engage in repetitive scrolling behaviour [44]. This connection is evident at the neuroanatomical level, with a reduction in the grey matter volume of the amygdala region [45]. Impulsivity is also recognised as a key factor in binge eating, leading to increased consumption of unhealthy foods [46]. An increase in impulsivity, coupled with a loss of self-control, is linked to the development of eating disorders [47]. This raises the question of whether the same underlying impulsivity that drives heightened social media use can also cause individuals to adopt unhealthy dietary patterns.

The findings of our study revealed a significant association between time spent on SPSM and specific dietary behaviours. Increased SPSM use was associated with greater consumption of sweetened drinks, energy drinks, fast food, and snacks. On the other hand, lower daily SPSM use was observed among participants with higher fruit and vegetable intake. This aligns with evidence that adherence to the Mediterranean diet, which is rich in fruits, vegetables, and whole grains, reduces impulsive behaviours and, consequently, internet addiction [48,49]. Our findings are consistent with a study from Saudi Arabia among young adults, which reported that frequent users of apps such as Snapchat, Instagram, and TikTok had higher consumption of fast food, sweets, and sugary drinks [12]. Another study among Brazilian adults also found that increased screen time was associated with a 21% higher likelihood of consuming unhealthy foods [50]. One possible reason for this is social media algorithms, which determine the content users are exposed to based on prior likes, shares, or comments [51]. Social media algorithms tend to promote engagement with similar, captivating content, while reducing exposure to health education posts [52].

Social media can influence dietary habits by conveying and promoting social norms [53]. Images of highly palatable, energy-dense foods that frequently appear in feeds are assumed to be common and socially approved choices, which affects users’ own dietary preferences and consumption patterns [54]. Robinson et al. also points out that both the type and amount of food people eat can be influenced by information about eating norms, indicating that normative cues could be used to promote healthier eating habits [54]. The marketing of food and beverages on social media by influencers has increased substantially, as they encourage consumers to like and share such products, thereby increasing their consumption. This reflects an involuntary exposure to persuasive digital marketing that can influence online behaviour and dietary patterns [55]. The adoption of unhealthy dietary habits is further worsened by persuasive app layouts and constant social media notifications, which diminish self-control and mindful eating [56].

However, our findings also indicate a weak to moderate association between SPSM and dietary habits, which should warrant particular attention when interpreting the results. In fact, the statistical significance of the associations found in the current study may mainly be attributed to the large sample size, which increased the ability to detect small effects; this is reflected in the values obtained when assessing the strength of the association was evaluated, yielding only weak to moderate associations. One possible explanation for the weak association reported in the current study is the type of SPSM material participants accessed, which was not assessed in this study because it was outside the study scope. However, the current results indicate that the primary reason for using SPSM was socialisation, with a small proportion of participants reporting dietary advice, health information, or education as their main reasons for using SPSM. This might explain the weaker associations observed in this study. Indeed, in a systematic review, Yu Wu et al. reported a positive correlation between certain social media content and food consumption, suggesting that the type of content accessed influences dietary behaviour [57].

Our study did not find a significant association between smartphone use and physical activity. This might be attributed to the prolonged use of smartphones recorded in the current study, which may have reduced the time available for practicing physical activity. However, a significant yet weak association was observed between social media use and physical activity. These findings align with previous research suggesting that physical activity levels are affected by many other factors, such as self-motivation, environmental conditions, and cultural norms [58]. Nonetheless, evidence indicates that some social media platforms have increased physical activity levels through digital programmes, demonstrating that content and context, rather than usage time, determine the relationship [59]. Although the association between social media and physical activity was statistically significant, the weak association suggests that this relationship warrants longitudinal investigation.

Considering the positive aspects of the digital food environment, recent studies suggest that SPSM can be utilised for health promotion by leveraging digital platforms for behavioural modification and public health interventions, because there is evidence supporting the efficacy of mobile-based nutrition education and health apps in improving dietary behaviours and physical activity [60,61,62]. An example is the development of the VegEze smartphone app to increase vegetable consumption among Australian adults, thereby promoting healthy dietary behaviour [63]. Thus, the prolonged SPSM usage time recorded in the current study underscores the critical need to reshape the digital food environment using SPSM to encourage healthy behaviours while reducing associated risks, thereby paving the way for successful public health initiatives in an increasingly digital world.

Strengths and Limitations: To the best of our knowledge, this is the largest household-based study with a robust sample size that examined the association and the strength of association between SPSM usage time, dietary habits, and physical activity levels in the UAE. The study employed a strong methodology and ensured that the results reflect the actual community-based diversity in terms of demographics, education, and socioeconomic status to minimise selection bias. The study not only focused on identifying associations with unhealthy food but also with healthy food. However, like all studies, this research has limitations. Its cross-sectional design allowed assessment of the associations between SPSM and dietary habits and physical activity, but it did not provide information about the direction of these associations. The association may be bidirectional, with participants engaging in unhealthy lifestyles, with poor dietary habits and low physical activity levels, may have been algorithmically exposed to similar social media content. Confounding factors that may independently affect screen time, dietary behaviours, and physical activity, such as sleep duration, psychological stress, or specific dietary interests, were not adjusted for, which may have affected the association. The study also did not take into account time spent on occupational purposes on the SPSM, as its scope was limited to recreational screen time. But the key confounding factors, age, gender, and socioeconomic status, were adjusted for.

Additionally, reliance on self-reported data on SPSM use may introduce recall bias. Participants might have underreported their susceptibility to social media influence, potentially introducing social desirability bias. Dietary habits were assessed through self-reported frequency of food intake, which may lead to under- or overreporting due to recall bias or social desirability bias, particularly because unhealthy food consumption may be socially less acceptable. The study did not evaluate the type of content that might have explained the weak association between SPSM and dietary behaviour and physical activity. Dietary habits were assessed by frequency rather than quantity, which may not accurately reflect participants’ eating patterns. For example, a person reporting regular fruit consumption might have eaten only small amounts, whereas another reporting less frequent intake may have consumed larger portions, resulting in misclassification bias. Although the results are representative of the UAE population, the country has many immigrants with diverse cultures, ethnicities, and lifestyles. This highlights opportunities for future research to explore behavioural differences across different groups.

Future Research and Implications: Our findings have helped identify the associations and the strength of associations between SPSM time, dietary behaviours, and physical activity levels among adults in the UAE. Given that the strength of association is weak to moderate, future research should investigate additional determinants and mediating pathways that better explain how SPSM influences dietary behaviours and physical activity. Analysing the types of content searched for on social networking sites such as Instagram, Facebook, and Twitter would be beneficial for understanding the underlying mechanisms driving the association. This could also help determine the extent to which dietary behaviour has been influenced by posts from social media influencers, friends, commercial advertisements, or promotional videos. Future research might also employ a longitudinal or experimental design to clarify the direction of the relationship and to account for the inclusion of other confounding factors. The study could also be expanded in the future to include other emirates of the UAE to better reflect the country’s cultural and lifestyle diversity.

The results of our study provide clinical insights by assisting nutritionists and clinicians in incorporating digital behaviour assessments into routine health evaluations and counselling. This enables more personalised and evidence-based nutrition advice by developing screening questionnaires, guided by our research, to assess patients’ SPSM engagement and dietary habits. Although the association is weak, public health professionals can use this information to design targeted interventions to reduce the negative impacts of the digital food environment and to promote critical evaluation of digital nutritional information. Educators and counsellors can integrate digital literacy into school and university programmes to recognise the influence of SPSM on dietary habits, as fostering such awareness from a young age is vital for establishing long-term, healthier behaviours. Furthermore, these findings underscore the importance of informing government officials and policymakers that, although the influence of SPSM on dietary behaviours may be moderate, incorporating awareness of digital use and nutrition education into public health initiatives can effectively promote healthier lifestyles among the digitally connected population in the UAE.

## 5. Conclusions

This study provides insight into the association and its strength between SPSM, dietary behaviours, and physical activity levels among adults in Abu Dhabi, UAE. The average time spent on SPSM was approximately 4 h per day, with socialising as the primary reason for personal use. There was a mainly highly significant association between SPSM and dietary behaviours and physical activity. Although the associations’ strength ranged from weak to moderate, the findings suggest that the digital food environment may influence dietary behaviours and physical activity. This implies that smartphones and social media can be effective platforms for nutrition interventions, providing a comprehensive, personalised, and engaging approach.

## Figures and Tables

**Table 1:** Characteristics of study participants (n = 5152).

Variable	Value
Age (years), Mean (SD)	36.4 (12.0)
Age Category, n (%)	
Young Adult (18–39)	3193 (62.0)
Middle (40–64)	1914 (37.2)
Older Adult (65 or older)	45 (0.9)
Sex, n (%)	
Male	1915 (37.2)
Female	3237 (62.8)
Body Mass Index (BMI) (kg/m^2^), Mean (SD)	27.2 (5.2)
BMI Classification, n (%)	
Underweight	132 (2.6)
Normal weight	1695 (32.9)
Overweight	1988 (38.6)
Obesity	1337 (26.0)
Nationality, n (%)	
Emirati	3027 (58.8)
Expats	2125 (41.2)
Health status (self-reported), n (%)	
Poor/Fair	239 (4.6)
Good	3522 (68.4)
Very Good/Excellent	1383 (26.8)
Marital Status, n (%)	
Single	1562 (30.3)
Married	3397 (66.0)
Divorced	108 (2.1)
Widow	69 (1.3)
Prefer not to say	16 (0.3)
Educational level, n (%)	
No school	160 (3.1)
Elementary	229 (4.4)
Intermediate	276 (5.4)
Secondary Stage	1477 (28.7)
College/University	2706 (52.5)
Master/Doctorate	304 (5.9)
Physical Activity, n (%)	
Low	3269 (63.4)
Moderate	1080 (21.0)
High	803 (15.6)
Smartphone Time (Min/Day), Mean (SD)	277.6 (165.6)
Smartphone Time Classification, n (%)	
Less than 2 h	598 (11.6)
2 to less than 4 h	1536 (29.8)
4 to less than 6 h	2009 (39.0)
6 h or more	1009 (19.6)
Social Media Time (Min/Day), Mean (SD)	234.6 (170.8)
Social Media Time Classification, n (%)	
Less than 2 h	1096 (21.3)
2 to less than 4 h	1784 (34.6)
4 to less than 6 h	1512 (29.3)
6 h or more	760 (14.8)

**Table 2:** The weekly frequency of food consumption reported by study participants (n = 5152).

Food Item	Never n (%)	1–2/wk. n (%)	3–4/wk. n (%)	5–6/wk. n (%)	Daily n (%)
Fruits	132 (2.6)	724 (14.1)	1415 (27.5)	592 (11.5)	2289 (44.4)
Vegetables	48 (0.9)	291 (5.6)	896 (17.4)	663 (12.9)	3254 (63.2)
Milk	944 (18.3)	1091 (21.2)	995 (19.3)	405 (7.9)	1717 (33.3)
Grains	30 (0.6)	81 (1.6)	241 (4.7)	380 (7.4)	4420 (85.8)
Meat	19 (0.4)	126 (2.4)	437 (8.5)	488 (9.5)	4082 (79.2)
Energy Drink	4042 (78.5)	615 (11.9)	275 (5.3)	76 (1.5)	144 (2.8)
Sweetened Beverage	1221 (23.7)	1704 (33.1)	1114 (21.6)	292 (5.7)	821 (15.9)
Fast foods	1469 (28.5)	2589 (50.3)	747 (14.5)	135 (2.6)	212 (4.1)
Snacks	457 (8.9)	883 (17.1)	825 (16.0)	292 (5.7)	2695 (52.3)
Breakfast	316 (6.1)	378 (7.3)	651 (12.6)	480 (9.3)	3327 (64.6)

**Table 3:** Average time spent on smartphones and social media (minutes/day) according to the weekly frequency of food consumption (n = 5152).

Food Item	Smartphone Time Mean (SD)					F-Value *	*p*-Value **
	Never	1–2/wk	3–4/wk	5–6/wk	Daily		
Fruits	296.7 (146.3)	284.7 (152.0)	308.1 (181.8)	290.3 (194.6)	255.8 (148.5)	9.697	<0.001
Vegetables	274.7 (127.2)	290.0 (136.5)	326.7 (199.0)	278.3 (181.4)	262.8 (151.6)	17.112	<0.001
Dairy	252.8 (138.6)	282.9 (154.5)	298.1 (187.3)	308.1 (204.9)	268.6 (159.4)	13.346	<0.001
Grains	284.2 (133.9)	246.5 (130.8)	307.8 (206.9)	272.7 (188.0)	276.8 (161.5)	2.325	0.054
Meat	253.4 (147.2)	241.4 (181.5)	268.9 (188.0)	274.5 (188.9)	280.1 (159.3)	3.575	0.006
Energy Drink	255.7 (143.9)	332.6 (193.7)	401.5 (221.0)	355.4 (201.9)	379.1 (235.2)	68.408	<0.001
Sweetened Beverage	213.4 (123.7)	276.7 (167.4)	300.8 (181.7)	280.5 (149.6)	342.3 (165.5)	64.224	<0.001
Fast foods	218.5 (145.9)	288.0 (162.3)	336.7 (175.5)	304.7 (152.8)	334.4 (180.3)	47.862	<0.001
Snacks	211.4 (124.4)	276.1 (176.3)	263.1 (175.7)	270.0 (159.4)	294.6 (162.2)	24.554	<0.001
Breakfast	290.4 (156.0)	281.6 (160.3)	285.1 (164.3)	275.5 (179.4)	274.7 (165.2)	0.227	0.924
	**Social Media Time** **Mean (SD)**					F-Value *	***p*** -Value **
	**Never**	**1–2/wk**	**3–4/wk**	**5–6/wk**	**Daily**		
Fruits	243.8 (146.5)	231.2 (149.6)	260.8 (189.4)	242.1 (202.9)	217.1 (154.3)	9.395	<0.001
Vegetables	234.4 (114.3)	246.9 (136.5)	284.1 (213.4)	231.8 (183.8)	220.5 (155.1)	17.070	<0.001
Dairy	193.1 (128.6)	245.6 (165.3)	254.8 (191.6)	262.7 (219.0)	232.2 (164.6)	22.320	<0.001
Grains	232.8 (139.9)	176.9 (122.2)	268.4 (216.1)	241.2 (199.4)	233.3 (165.9)	4.262	0.002
Meat	174.5 (117.3)	205.0 (165.6)	233.3 (206.2)	232.8 (192.3)	236.2 (164.1)	1.893	0.109
Energy Drink	210.3 (145.6)	292.8 (199.2)	377.3 (234.5)	335.0 (232.5)	344.6 (244.2)	91.132	<0.001
Sweetened Beverage	168.0 (121.9)	230.5 (175.2)	254.2 (182.4)	238.1 (149.6)	314.7 (174.9)	82.174	<0.001
Fast foods	174.4 (142.0)	246.0 (172.4)	296.0 (181.4)	255.7 (160.1)	283.7 (178.2)	51.585	<0.001
Snacks	164.4 (115.6)	246.1 (186.9)	221.4 (172.1)	229.8 (153.5)	247.4 (171.4)	22.210	<0.001
Breakfast	215.9 (139.3)	233.6 (157.9)	237.2 (160.8)	229.4 (175.8)	236.8 (171.9)	3.576	0.006

SD: Standard Deviation. * F value from ANCOVA and adjusted for the covariates age, BMI, sex, and monthly income. ** *p*-value was based on the ANCOVA analysis at a 5% level of significance.

**Table 4:** Association between the categories of time spent on smartphones and social media with categories of weekly frequency of food consumption and physical activity (n = 5152).

Variable	Smartphone				Social Media			
	*X^2^*	*p*-Value	Cramer’s V	r	*X^2^*	*p*-Value	Cramer’s V	r
Dietary								
Fruits	53.383	<0.001	0.059	−0.074 *	72.259	<0.001	0.068	−0.057 *
Vegetables	70.320	<0.001	0.067	−0.080 *	77.615	<0.001	0.071	−0.069 *
Dairy	86.110	<0.001	0.074	0.002	122.874	<0.001	0.089	0.033 *
Grains	16.988	0.150	0.033	0.015	31.418	0.002	0.045	0.018
Meat	45.405	<0.001	0.054	0.066 *	53.544	<0.001	0.055	0.055 *
Energy Drink	220.790	<0.001	0.120	0.166 *	309.643	<0.001	0.142	0.181 *
Sweetened Beverage	461.929	<0.001	0.173	0.212 *	619.416	<0.001	0.200	0.232 *
Fast foods	405.021	<0.001	0.162	0.218 *	401.854	<0.001	0.161	0.220 *
Snacks	149.168	<0.001	0.098	0.130 *	145.090	<0.001	0.097	0.096 *
Breakfast	22.313	0.034	0.038	−0.021	41.025	<0.001	0.052	0.008
Physical activity	8.640	0.195	0.029	0.001	16.370	0.012	0.040	−0.024

* Kendall’s tau-b correlation is significant, *p* < 0.05.

**Table 5:** Association of smartphone and social media main usage purpose (only one response was allowed) with gender (n = 5152).

Main Use Purpose	Total (n = 5152) n (%)	Male (n = 1915) n (%)	Female (n = 3237) n (%)	*X* ^2^	*p*-Value	Cramer’s V
Smartphone				97.853	<0.001	0.138
Education/information	422 (8.2)	122 (6.4)	300 (9.3)			
E-mail	132 (2.6)	72 (3.8)	60 (1.9)			
Social networking services	3692 (71.7)	1301 (67.9)	2391 (73.9)			
Games	178 (3.5)	104 (5.4)	74 (2.3)			
Movie/video/music	523 (10.2)	250 (13.1)	273 (8.4)			
Online shopping	31 (0.6)	8 (0.4)	23 (0.7)			
Others	174 (3.4)	58 (3.0)	116 (3.6)			
Social media				91.131	<0.001	0.133
News	392 (7.6)	203 (10.6)	189 (5.8)			
Education/information	319 (6.2)	109 (5.7)	210 (6.5)			
Social Networking Services	3854 (73.8)	1451 (75.8)	2403 (74.2)			
Health advice/information	95 (1.8)	22 (1.1)	73 (2.3)			
Dietary advice/information	32 (0.6)	8 (0.4)	24 (0.7)			
Shopping	144 (2.8)	16 (0.8)	128 (4.0)			
Others	316 (6.1)	106 (5.5)	210 (6.5)			

## Data Availability

The data used in this study is available from the corresponding author on reasonable request.
